# A high-entropy alloy with hierarchical nanoprecipitates and ultrahigh strength

**DOI:** 10.1126/sciadv.aat8712

**Published:** 2018-10-12

**Authors:** Zhiqiang Fu, Lin Jiang, Jenna L. Wardini, Benjamin E. MacDonald, Haiming Wen, Wei Xiong, Dalong Zhang, Yizhang Zhou, Timothy J. Rupert, Weiping Chen, Enrique J. Lavernia

**Affiliations:** 1 Guangdong Key Laboratory for Advanced Metallic Materials Processing, South China University of Technology, Guangzhou, Guangdong 510640, China.; 2 Department of Materials Science and Engineering, University of California, Irvine, CA 92697, USA.; 3 Materials and Structural Analysis, Thermo Fisher Scientific, Hillsboro, OR 97124, USA.; 4 Department of Materials Science and Engineering, Missouri University of Science and Technology, Rolla, MO 65409, USA.; 5 Department of Mechanical Engineering and Materials Science, University of Pittsburgh, Pittsburgh, PA 15261, USA.

## Abstract

High-entropy alloys (HEAs) are a class of metallic materials that have revolutionized alloy design. They are known for their high compressive strengths, often greater than 1 GPa; however, the tensile strengths of most reported HEAs are limited. Here, we report a strategy for the design and fabrication of HEAs that can achieve ultrahigh tensile strengths. The proposed strategy involves the introduction of a high density of hierarchical intragranular nanoprecipitates. To establish the validity of this strategy, we designed and fabricated a bulk Fe_25_Co_25_Ni_25_Al_10_Ti_15_ HEA to consist of a principal face-centered cubic (fcc) phase containing hierarchical intragranular nanoprecipitates. Our results show that precipitation strengthening, as one of the main strengthening mechanisms, contributes to a tensile yield strength (σ_0.2_) of ~1.86 GPa and an ultimate tensile strength of ~2.52 GPa at room temperature, which heretofore represents the highest strength reported for an HEA with an appreciable failure strain of ~5.2%.

## Teaser

HEAs’ compositional space allows the design of hierarchical microstructures that contribute to the exceptional strength values.

## INTRODUCTION

The traditional alloy design concept combines one or two principal elements with additional minor elements, an approach that restricts the development and application of alloys due to the limited compositional space explored (*1*). Thus, to enable the pursuit of alloys with special microstructures and properties, such as high strength, new design concepts are needed. Recently, a brand new class of metallic materials that consists of at least five principal elements, each contributing 5 to 35 atomic % (at %) referred to as multi-principal element alloys or high-entropy alloys (HEAs), has attracted worldwide attention (*2*, *3*). HEAs have unique combinations of properties that are not attainable in conventional alloys, including high strength and hardness, excellent resistance to high-temperature softening, unique magnetic properties, superior corrosion and oxidation resistance, strong fatigue resistance, attractive tribological properties, good creep resistance, excellent irradiation tolerance, high thermal stability, and superior mechanical performance at cryogenic temperatures (*2*, *4*–*11*).

HEAs have simple random solid solutions rather than ordered and/or intermetallic phases; partly due to a high entropy of mixing (high-entropy effect), which notably reduces the Gibbs free energy of mixing (*2*, *3*). Solid-solution phases reported in HEAs generally include five types of phases; that is, disordered face-centered cubic (fcc, A1), disordered body-centered cubic (bcc, A2), ordered fcc (L1_2_), ordered bcc (B2), and hexagonal close-packed (hcp, A3) (*2*, *12*, *13*). HEAs can be classified as single- and multiphase HEAs. Despite the maximized configurational entropy of mixing in single-phase HEAs compared to those of multiphase HEAs, single-phase HEAs usually exhibit disappointing mechanical property combinations. For instance, single-phase fcc-structured HEAs show outstanding ductility with low strength (*14*), and single-phase bcc-structured HEAs have high strength but low ductility (*15*). Inspection of the published literature reveals that a number of strategies have emerged in an effort to design multiphase HEAs that contain both ductile and strong phases (*16*, *17*). Here, we report on the design and fabrication of an Fe_25_Co_25_Ni_25_Al_10_Ti_15_ HEA that contains a ductile fcc matrix with a small volume fraction of a strong interdispersed bcc phase.

Different strengthening mechanisms, such as phase transformation strengthening, solid-solution strengthening, dislocation strengthening, grain-boundary strengthening, precipitation strengthening, and load transfer via the introduction of strong phases, can be used to achieve high strength/hardness in multiphase HEAs (*18*–*30*). Of these, load transfer and precipitation strengthening are considered to be the two most effective strengthening mechanisms. Excessive amounts of strong phases lead to a notable decrease in ductility or even failure before yielding during mechanical testing (*25*). Precipitates are often observed in HEAs when a significantly positive enthalpy of mixing (or significantly negative enthalpy of formation of intermetallic phases) cannot be counteracted by a high entropy of mixing (*17*, *27*, *28*). A dispersion of coherent γ′ precipitates in the γ matrix in some Al- and/or Ti-containing HEAs leads to high strength/hardness at room temperature and at high temperatures (*17*, *27*, *28*, *31*).

Precipitates in HEAs usually contain multiple principal elements (*27*, *28*). Two interesting questions arise: (i) Can a hierarchical architecture involving nanoprecipitates be formed in HEAs; that is, can some secondary nanoprecipitates form inside these primary nanoprecipitates consisting of multi-principal elements? (ii) Can an ultrahigh strength be achieved as a result of the high density of intragranular nanoprecipitates with such a hierarchical architecture? Here, we design a novel Fe_25_Co_25_Ni_25_Al_10_Ti_15_ HEA based on the fcc single-phase equiatomic FeCoNi medium-entropy alloy in which two elements, Al and Ti, are added (*14*). Al and Ti tend to form intermetallic phases with Fe, Ni, and Co, which have remarkably high mutual solubility with each other, and accordingly, the addition of Al and Ti into an FeCoNi alloy is anticipated to form a high density of intragranular nanoprecipitates having multiple principal elements. To ensure the formation of stabilized solid-solution phases without additional complex phases, empirical conditions of Ω ≥ 1.1 and δ ≤ 6.6% should be satisfied at minimum, where Ω is an indicator of the stability of the multicomponent solid solution and δ is the atomic size difference of the component elements (*32*, *33*). In the meantime, Ω infinitely approaching the threshold value of 1.1 would potentially lead to the formation of secondary precipitates. Thus, the chemical composition of Fe_25_Co_25_Ni_25_Al_10_Ti_15_ with Ω = 1.119 and δ = 6.277 was designed from this concept (see the supplementary materials for more details on the design strategy). Thus far, bulk HEAs have been predominantly fabricated by casting, during which phase separation may occur. Here, we synthesize a bulk Fe_25_Co_25_Ni_25_Al_10_Ti_15_ HEA by mechanical alloying (MA) followed by consolidation via spark plasma sintering (SPS). We show that a bulk Fe_25_Co_25_Ni_25_Al_10_Ti_15_ HEA exhibits a primary fcc phase with a small amount of bcc phase based on x-ray diffraction (XRD). The fcc matrix (γ) is reinforced by a very high density of intragranular nanoprecipitates (γ′) in which significantly finer secondary nanoprecipitates (γ*) are present. Note that the symbol γ* designated herein is only used to differentiate γ′ and γ* nanoprecipitates and to show the relationship between γ′ and γ* nanoprecipitates. The γ, γ′, and γ* phases exhibit A1, L1_2_, and A1 structures with very similar lattice parameter, respectively. The high density of hierarchical intragranular nanoprecipitates in the bulk Fe_25_Co_25_Ni_25_Al_10_Ti_15_ HEA results in an ultrahigh strength, one of the highest strengths reported to date for HEAs. The results reported in our study provide important insight into design strategies that can be effectively used to achieve ultrahigh strength in HEAs or other multicomponent alloys.

## RESULTS

### XRD and electron backscatter diffraction studies

**Fig. 1:**
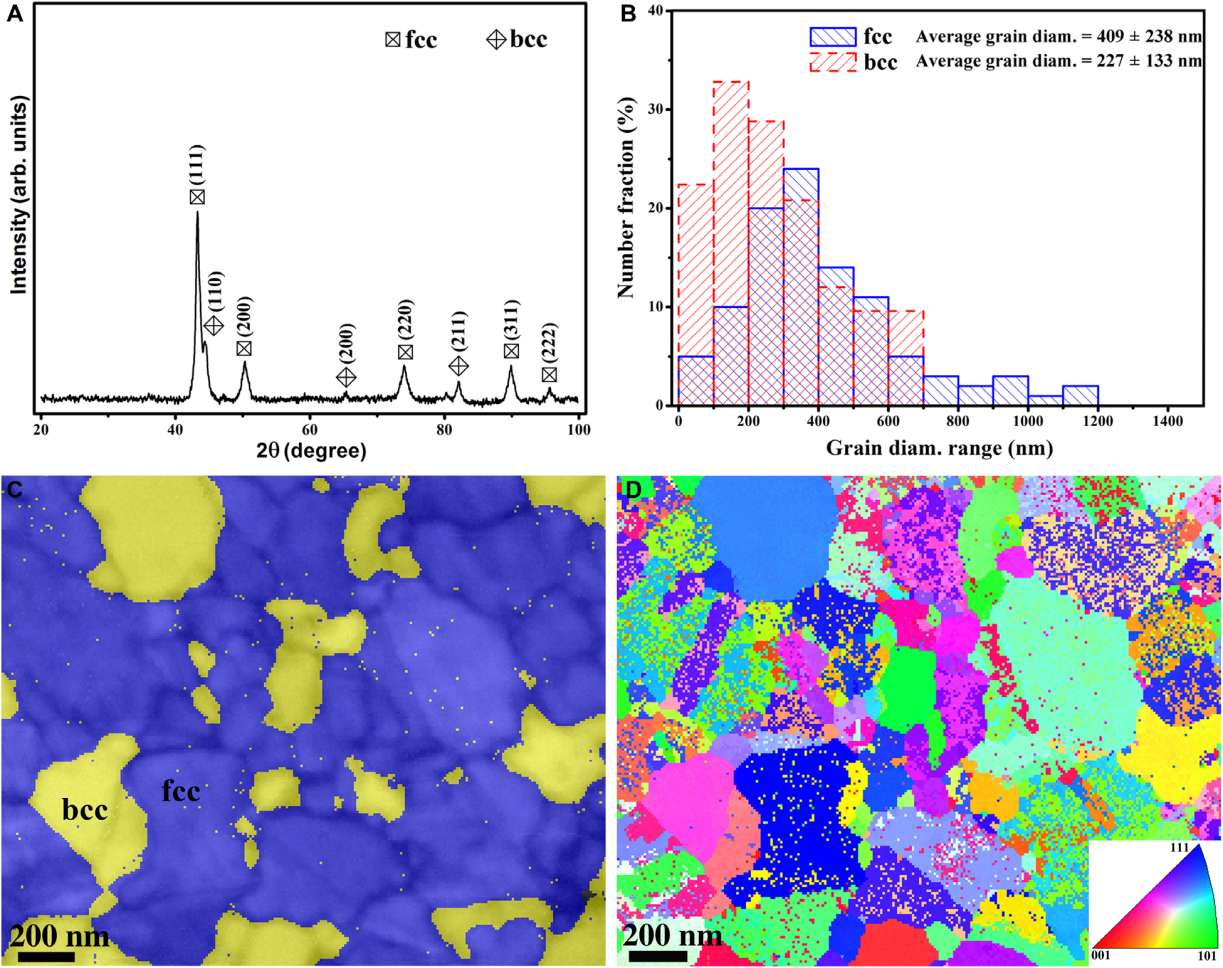
X-ray scattering and EBSD analyses of the bulk Fe_25_Co_25_Ni_25_Al_10_Ti_15_ HEA. (**A**) XRD pattern. (**B**) Statistics of grain diameters for the two phases were collected on the basis of EBSD and TEM images. (**C**) EBSD phase map. (**D**) EBSD IPF corresponding to the EBSD phase map in (C).

As shown in Fig. 1A, the XRD pattern reveals that the bulk Fe_25_Co_25_Ni_25_Al_10_Ti_15_ HEA is composed of a primary fcc phase (~82.3 volume %) and a small amount of bcc phase (~17.7 volume %), which is confirmed by the electron backscatter diffraction (EBSD) phase map in Fig. 1C. The EBSD inverse pole figure (IPF) in Fig. 1D corresponding to the EBSD phase map in Fig. 1C indicates that, inside the fcc grains, there are some unidentified phases, which correspond to a high density of hierarchical intragranular nanoprecipitates, as we will show hereinafter. Note that there are no precipitates in the bcc phase. On the basis of numerous transmission electron microscopy (TEM) images and EBSD maps, grain diameters of the fcc phase and of the bcc phase were measured. The statistical grain diameter distribution, presented in Fig. 1B, indicates that the diameters of the fcc grains range from several nanometers to 1200 nm, while those of the bcc grains are in the range of several nanometers to 700 nm. In addition, average diameters for the fcc phase and for the bcc phase are ~409 and ~227 nm, respectively.

### Microstructures

**Fig. 2:**
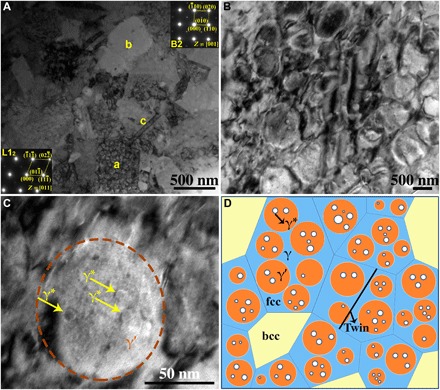
Microstructure of the bulk Fe_25_Co_25_Ni_25_Al_10_Ti_15_ HEA. (**A**) Bright-field TEM image and SAED patterns corresponding to grains a and b. (**B**) High density of γ′ nanoprecipitates inside the fcc phase. (**C**) Bright-field TEM image of a γ′ nanoprecipitate showing some secondary γ* (as indicated by the arrows) nanoprecipitates inside. (**D**) Schematic diagram shows the microstructure of the alloy, indicating that fcc γ matrix grains (blue) have hierarchical intragranular precipitates, i.e., primary γ′ precipitates (orange) and secondary γ* precipitates (white), and that there are some twins in the fcc grains.

In the bright-field TEM image shown in Fig. 2A, the selected-area electron diffraction (SAED) pattern of the [011] zone axis corresponding to grain a presented in the lower left inset exhibits reflections of an ordered fcc (L1_2_ structure), and the SAED pattern of the [001] zone axis corresponding to grain b presented in the upper right inset exhibits reflections of an ordered bcc (B2 structure). Evidently, a high density of nanoprecipitates can be seen in grain a. Figure 2B shows that high densities of γ′ nanoprecipitates are present inside the fcc phase. In addition, the scanning TEM (STEM) image of grain I in fig. S1 further confirms the presence of nanoprecipitates inside the fcc grains. Figure 2C shows the presence of some secondary γ* precipitates (as indicated by arrows), several nanometers to tens of nanometers in size, in the γ′ precipitate. Also, there are twins present in some fcc grains such as grain c in Fig. 2A. In addition, fig. S2 confirms the presence of twins in additional fcc grains. It is not surprising that twinning can occur in these grains, which may arise from the nature of fcc-structured phases in HEAs, which usually exhibit low stacking fault energy (*11*, *13*, *30*). It is argued that the twins observed in the sintered HEAs are likely deformation twins retained from milled powders and/or annealing twins generated during the SPS process (*34*). Note that precipitates or twins were not observed in the bcc phase. A schematic diagram of the microstructure of the bulk Fe_25_Co_25_Ni_25_Al_10_Ti_15_ HEA is illustrated in Fig. 2D, showing that the fcc phase (γ, blue) contains hierarchical intragranular precipitates, i.e., the primary γ′ precipitates (orange) and the secondary γ* precipitates (white); however, there are no precipitates in the bcc grains (yellow).

Figure S1C shows the statistical distribution of the spacing between two adjacent γ′ nanoprecipitates, which ranges from several nanometers to ~45 nm, and the statistical γ′ nanoprecipitate diameter distribution ranging from ~20 to ~100 nm. Estimated average values of the γ′ nanoprecipitate’s diameter and the spacing between adjacent γ′ nanoprecipitates are 57 and 11 nm, respectively. In addition, the estimated volume fraction of γ′ nanoprecipitates in the fcc phase is ~62 volume %, and therefore, the total volume fraction of γ′ nanoprecipitates in the bulk Fe_25_Co_25_Ni_25_Al_10_Ti_15_ HEA is ~51 volume %.

Chemical compositions of the phases in the bulk Fe_25_Co_25_Ni_25_Al_10_Ti_15_ HEA are listed in table S1. Chemical compositions of the bcc phase and fcc phase (γ + γ′ + γ*) were obtained by energy dispersive spectroscopy (EDS) in TEM, whereas chemical compositions of the fcc γ matrix and γ′ precipitates were measured by EDS in STEM. The bcc phase is an Al-Ti–rich phase, and the fcc phase (γ + γ′ + γ*) is an Al-Ti–depleted phase, which is consistent with high contents of Al and Ti favoring the formation of bcc-structured phases in HEAs (*8*, *12*, *16*). Furthermore, the fcc γ matrix is an Fe-(Co,Ni)–based solid-solution phase with some Al and Ti, and the γ′ precipitate is a (Ni,Co)_3_-(Ti,Al)–based intermetallic phase with some Fe present. Note that the chemical composition of γ* precipitates has not been obtained because of their very small sizes.

### Mechanical properties

**Fig. 3:**
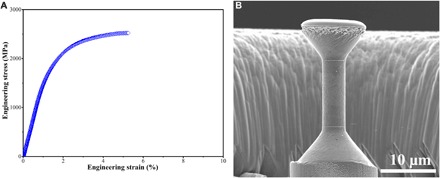
In situ SEM microtensile testing of the bulk Fe_25_Co_25_Ni_25_Al_10_Ti_15_ HEA. (**A**) Representative tensile engineering stress-strain curve of the SPS-consolidated sample at room temperature. (**B**) Corresponding tensile coupon having a cylindrical gauge section with a 4-μm diameter and a 12-μm length between electron beam–deposited Pt reference markers.

In situ scanning electron microscopy (SEM) microtensile testing was used to measure the mechanical behavior of the consolidated Fe_25_Co_25_Ni_25_Al_10_Ti_15_ HEA at room temperature. A representative tensile engineering stress-strain curve and its corresponding tensile coupon are shown in Fig. 3 (A and B). The tensile yield strength (σ_0.2_, calculated at a 0.2% offset strain), ultimate tensile strength, and elongation at failure are ~1860 MPa, ~2520 MPa, and ~5.2%, respectively. It is obvious that the tensile stress-strain curve reveals significant plastic strain for such a high-strength material, accompanied by work hardening.

## DISCUSSION

We first analyze the origin of the ultrahigh strength of the newly developed Fe_25_Co_25_Ni_25_Al_10_Ti_15_ HEA at room temperature. We hypothesize that the ultrahigh strength is primarily attributed to precipitation strengthening generated from the high density of hierarchical intragranular nanoprecipitates in the primary fcc phase.

**Fig. 4:**
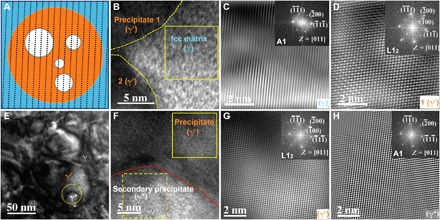
HRTEM images of the fcc phase containing hierarchical intragranular nanoprecipitates. (**A**) Schematic diagram of coherently hierarchical nanoprecipitates in the fcc phase. (**B**) HRTEM image of the fcc γ matrix and two γ′ precipitates. (**C**) IFFT of the square area (the γ matrix) in (B) with corresponding FFT presented in the inset. (**D**) IFFT of the γ′ precipitate 1 in (B) with corresponding FFT presented in the inset. (**E**) Bright-field TEM image of the γ, γ′, and γ* phases. (**F**) HRTEM image of the circled area in (E). (**G**) IFFT of the square area indicated by solid line (the γ′ precipitate) in (F) with corresponding FFT presented in the inset. (**H**) IFFT of the square area indicated by dashed lines (the γ* precipitate) in (F) with corresponding FFT in the inset.

Precipitation strengthening occurs by two mechanisms: the shearing mechanism or the Orowan bypass mechanism, depending on many factors, including the precipitate size, coherency, antiphase boundary (APB) energy, and strength (or hardness) of the precipitate (*35*). We first discuss crystal structures and coherency of the fcc γ matrix, the primary γ′ precipitate, and the secondary γ* precipitate in the fcc phase. Figure 4 shows high-resolution TEM (HRTEM) images of the fcc phase containing hierarchical intragranular nanoprecipitates. Figure 4A displays a schematic diagram of the detailed microstructure of coherently hierarchical nanoprecipitates in the fcc phase, where dashed lines in Fig. 4A denote that the γ matrix, γ′ precipitates, and γ* precipitates are coherent, which will be discussed hereinafter. An HRTEM image containing the γ matrix and two γ′ precipitates (marked as precipitates 1 and 2, respectively) inside the fcc grain is shown in Fig. 4B. Figure 4C shows the inverse fast Fourier transform (IFFT) of the square area in the γ matrix (Fig. 4B), with its corresponding FFT presented in the inset, and the FFT corresponds to an SAED pattern along the [011] zone axis of a disordered fcc (A1) structure with a lattice parameter of ~3.601 Å. The IFFT of the γ′ precipitate 1 in Fig. 4B is displayed in Fig. 4D with corresponding FFT presented in the inset; the FFT corresponds to an SAED pattern along the [011] zone axis of an ordered fcc (L1_2_) structure with a lattice parameter of ~3.605 Å. This suggests that the γ matrix and the γ′ precipitate have an orientation relationship of <011>_γ_∥ <011>_γ′_ and {111}_γ_∥ {111}_γ′_. Figure 4E presents a bright-field TEM image of the γ, γ′, and γ* phases, and an HRTEM image of the circled area in Fig. 4E is shown in Fig. 4F, which reveals the interface between γ′ and γ* precipitates. Similarly, as shown in Fig. 4G, the FFT of the γ′ precipitate suggests an ordered L1_2_ structure with a lattice parameter of ~3.605 Å; however, Fig. 4H shows that the FFT of the γ* precipitate suggests a disordered A1 structure with a lattice parameter of ~3.601 Å. Thus, γ′ and γ* precipitates also have an orientation relationship of <011>_γ′_∥ <011>_γ*_ and {111}_γ′_∥ {111}_γ*_. It is therefore concluded that the γ matrix, γ′ precipitate, and γ* precipitate are coherent, and they exhibit an orientation relationship of <011>_γ_∥ <011>_γ′_∥ <011>_γ*_ and {111}_γ_∥ {111}_γ′_∥ {111}_γ*_. This orientation relationship is also further confirmed in fig. S3. The γ matrix and the γ* precipitate have a disordered A1 structure, while the γ′ precipitate displays an ordered L1_2_ structure, with approximately identical lattice parameters. Hence, the γ, γ′, and γ* phases exhibit very similar crystal structures and have almost identical lattice parameters, which explains why XRD can only identify one fcc crystal structure, as is the cases in many HEAs (*12*, *13*, *17*). In summary, we conclude that the fcc γ matrix is an A1-structured Fe-(Co,Ni)–based solid-solution phase, the γ′ precipitate is a L1_2_-structured (Ni,Co)_3_-(Ti,Al)–based intermetallic phase, and the γ* precipitate is an A1-structured phase with an undetected composition.

The shearing mechanism will be active when a precipitate with small size is coherent, whereas the Orowan bypass mechanism occurs preferentially when the precipitate is incoherent (*27*, *28*, *35*). Since γ′ and γ* precipitates are coherent with the γ matrix and γ′ precipitates have an average diameter of ~57 nm, shearing is suspected to be the operative mechanism. To obtain direct evidence of this mechanism, in situ compression tests were carried out in the TEM at room temperature. The recorded test (movie S1) reveals that dislocations do in fact shear γ′ precipitates. Figure S4 presents sequential snapshots from the movie that track the motion of dislocations with arrows. The dislocations first generated in the γ matrix propagate through the γ′ precipitates, which implies that shearing of the precipitate has occurred as opposed to the Orowan bypass mechanism. As dislocations shear the precipitate, the dislocation motion is hindered, leading to accumulation of dislocations in γ′ precipitates. It is expected that dislocations also shear coherent γ* precipitates as they traverse the γ′ precipitate. The observation of the abovementioned dynamic process during the in situ TEM compression test suggests that the shearing mechanism is also active in room temperature tensile testing. Hence, it is anticipated that a relatively high stress will be needed for the shearing mechanism of both of γ′ and γ* precipitates, thereby leading to an exceptional precipitation strengthening. In addition to precipitation strengthening, other strengthening mechanisms are believed to contribute to the ultrahigh strength measured. Because of submicrometer grain sizes, 409 and 227 nm for the fcc and bcc phase, respectively, grain-boundary strengthening is likely to occur (*23*, *34*). Furthermore, the bcc phase with ordered B2 structure is usually considered as a hard/strong phase that can result in a high increment in yield strength due to a load transfer mechanism (*13*, *36*).

**Fig. 5:**
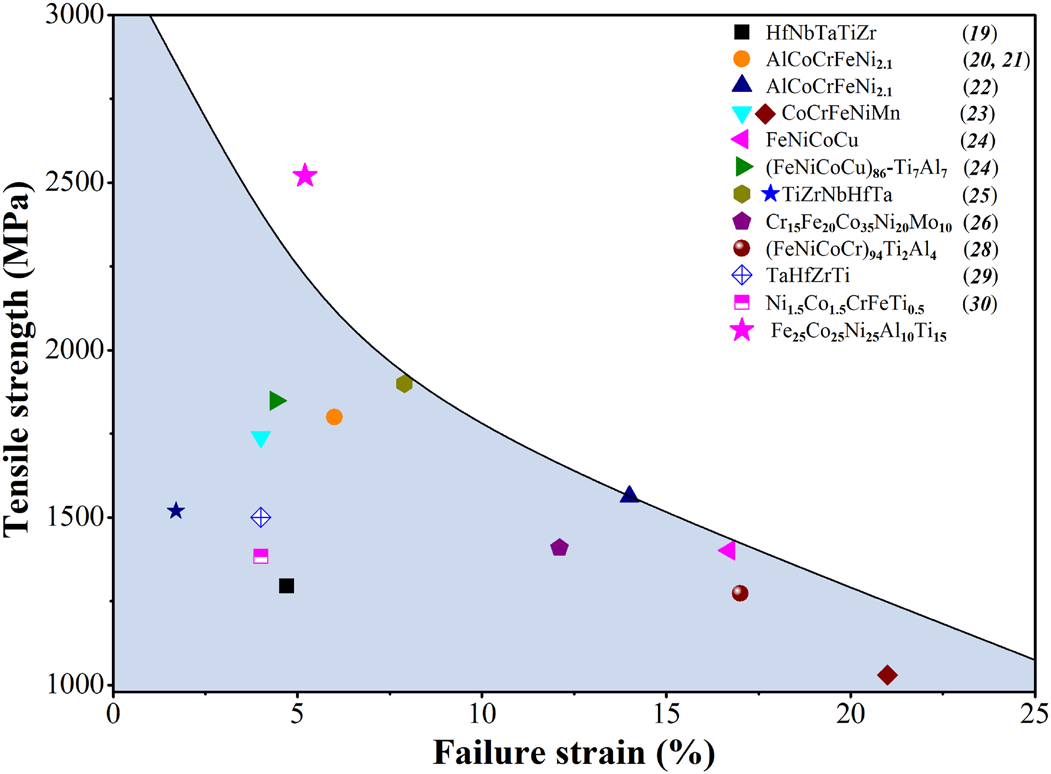
Tensile strength-failure strain plot of selected HEAs. It reveals that the bulk Fe_25_Co_25_Ni_25_Al_10_Ti_15_ HEA shows the highest tensile strength in comparison with available literature data for HEAs having high tensile strength.

To provide insight into the strength of the material presented in this work, the tensile strength versus failure strain is plotted in Fig. 5 alongside other HEAs that exhibit high strength (yield strength and tensile strength both greater than 1 GPa) (*19*–*30*). The performance of these HEAs is attributed to complex microstructures achieved by various processing routes outlined in table S2. Note that most of reported HEAs have yield strengths and tensile strengths less than 1 GPa, falling outside the range of mechanical performance presented in Fig. 5. Specifically, for HEAs processed by cold rolling/cryo rolling and high-pressure torsion, high strengths are mainly attributed to dislocation strengthening and grain-boundary strengthening, as well as the presence of strong B2 and/or σ phases (*19*–*25*). Grain-boundary strengthening and dispersion strengthening mainly account for the strengthening mechanisms of the Ni_1.5_Co_1.5_CrFeTi_0.5_ HEA with fine grains and oxide contaminants (*30*). The high strength of the as-cast bcc TaHZrTi refractory HEA is due to the high-load transfer ability of bcc refractory HEAs (*29*). The strengthening mechanism in the precipitation-strengthened HEAs is dominated by the shearing mechanism (*26*, *28*). The uniqueness of precipitation strengthening in the Fe_25_Co_25_Ni_25_Al_10_Ti_15_ HEA is due to the hierarchical precipitate spatial distribution, which is not found in precipitation-strengthened HEAs reported in the literature. In previously reported HEAs, dislocations only need to shear coherent γ′ precipitates (*26*, *28*). Here, dislocations must shear both the γ′ (primary precipitates) and the γ* (secondary nanoprecipitates). γ* nanoprecipitates act as an additional obstacle, which results in an exaggerated precipitation strengthening effect. As shown in Fig. 5, compared to data reported in the literature, the Fe_25_Co_25_Ni_25_Al_10_Ti_15_ HEA exhibits the highest tensile strength with an appreciable failure strain (~5.2%) at room temperature, suggesting that the proposed strategy in this work is valid due to the strengthening mechanisms previously discussed.

Upon inspection of the fracture surface generated from in situ SEM microtensile testing (fig. S5), both faceted and dimpled regions are present, corresponding to brittle and ductile failure, respectively. Evidently, plastic deformation of the alloy occurs primarily through the ductile fcc phase, which has the aforementioned hierarchical architecture. Dislocation accumulation/storage is enhanced inside the fcc grains due to the presence of hierarchical intragranular nanoprecipitates, which may result in enhanced strain hardening and improved ductility (*37*) relative to other reported HEAs. This proposed mechanism accounts for the appreciable work hardening and failure strain of ~5.2% exhibited by the Fe_25_Co_25_Ni_25_Al_10_Ti_15_ HEA under tension at room temperature.

Hierarchical intragranular nanoprecipitates in HEAs were attained through MA followed by SPS. The synthesis of HEAs by MA results in remarkably extended solid solubility, and therefore, supersaturated solid solutions may be formed in the as-milled HEA powders (*30*, *34*). Subsequently, the formation of more equilibrated phases occurs during consolidation at elevated temperature (*12*, *30*, *34*). The 49-hour milled Fe_25_Co_25_Ni_25_Al_10_Ti_15_ HEA powders are supersaturated solid solutions consisting of a primary bcc phase and a small amount of fcc phase (see fig. S6). However, following SPS, the bulk sample exhibits a primary fcc phase (containing hierarchical intragranular nanoprecipitates) and a minor bcc phase. The primary γ′ nanoprecipitates have multiple principal elements; they are (Ni,Co)_3_-(Ti,Al)–based phase and may have some other constituent elements such as Fe. As a result, some secondary γ* nanoprecipitates precipitate from the primary γ′ nanoprecipitates during the late stage of sintering. The γ* nanoprecipitates may form through phase separation mechanism during the cooling process of SPS, which can be understood by performing Scheil-Gulliver nonequilibrium modeling, as shown in fig. S7 (*38*, *39*). It is not uncommon to observe nanoscale phase separation upon cooling of HEAs, since the diffusion of some species in HEAs may be limited, and thus promoting nanoscale phase separation within the matrix (*12*, *31*, *36*). The nonequilibrium thermodynamic modeling indicates that, along with the major species, Fe is within the fcc-structured γ* phase, which is consistent with the EDS analysis.

In summary, the current work demonstrates that it is possible to design a multiphase Fe_25_Co_25_Ni_25_Al_10_Ti_15_ HEA, having a high density of hierarchical intragranular nanoprecipitates, with ultrahigh strength; the values reported herein represent one of the highest strength values ever reported for any HEAs.

## MATERIALS AND METHODS

### Materials processing

The bulk Fe_25_Co_25_Ni_25_Al_10_Ti_15_ samples were synthesized via MA followed by SPS. A high-energy planetary ball-milling machine was used to perform the MA process. Blended elemental powders of Fe, Co, Ni, Al, and Ti (purity of >99.7 weight % and particle sizes of ≤45 μm), and tungsten carbide balls were placed in a stainless steel vial. The entire MA process was operated at 300 rpm and protected by argon. First, the powders were subjected to dry milling without a process control agent (PCA) for 45 hours. Four hours of wet milling was conducted using ethanol as PCA following 45 hours of dry milling. Then, the 49-hour as-milled powders were dried. Subsequently, sieved powders with particle sizes of ≤75 μm were obtained after the dried powders passed through a 75-μm sieve. A Dr. Sinter 825 apparatus (Sumitomo Coal Mining Co. Ltd., Japan) was applied to consolidate the sieved powders into bulk samples at 1000°C. During SPS, the vacuum pressure was maintained at <8 Pa, and a constant pressure of 30 MPa was used with a heating rate of 90°C/min. Following SPS, SPS-sintered discs with sizes of Ø20 mm by ~8 mm were obtained, and specimens for subsequent tests were cut by electrical discharge machining.

### Microstructural characterization

A Bruker D8 ADVANCE x-ray diffractometer with a Cu Kα radiation was used to analyze the powders and bulk samples. EBSD was performed using an Oxford Instruments Nordlys Nano detector that is equipped to a Phillips XL-30 SFEG SEM. An Oxford Channel 5 software was adopted to analyze the EBSD data. In addition, a JEOL JEM-2100 (200 kV) and a JEOL JEM-2500SE (200 kV) TEM with SAED and with EDS were used to observe the microstructure and to obtain chemical compositions of the Fe_25_Co_25_Ni_25_Al_10_Ti_15_ HEA. TEM specimens were first polished to ~20 μm and then thinned to electron transparency by ion milling.

### In situ SEM microtensile testing

Microtensile coupons were prepared by focused ion beam (FIB) on an FEI Quanta 3D dual beam (SEM/FIB) using a semiautomated “lathe-milling” procedure described in detail in (*40*). The resulting microtensile coupons have a cylindrical gauge section with an average diameter of 4 μm and a length of 12 μm.

Uniaxial tension tests were performed under electron beam observation using a nanomechanical testing system (model FT-NMT03, FemtoTools, Buchs, Switzerland) at room temperature in displacement-controlled mode with a nominal strain rate of 1 × 10^−3^ s^−1^. The force was measured by a micro-electro-mechanical system (MEMS)–based microforce sensor (model FT-S200’000), whose 50-μm by 50-μm flat probe tip was milled into a tensile grip by FIB (see fig. S8). A sample displacement was measured simultaneously using a piezo-based actuation system with subnanometer resolution. Platinum reference markers were electron beam deposited on both ends of the gauge section to enable relative displacement measurements in post processing. Marker positions were tracked frame-by-frame using a custom MATLAB script to calculate the sample elongation.

### In situ compression testing in TEM

A FEI Quanta 3D dual beam (SEM/FIB) system was used to prepare pillars with the geometry of 1500 nm by 850 nm by 120 nm. In situ TEM compression tests were conducted at room temperature using a Hysitron picoindenter 95 equipped with a flat tip in a JEOL JEM-2800 TEM operating at 200 kV. The uniaxial compression tests were performed in displacement-controlled mode with an axial displacement rate of 3 nm s^–1^. A Gatan one-view charge-coupled device camera was used to record time-resolved TEM images of the regions of interest at 30 frames per second.

### Computational methods

The CALPHAD (CALculation of PHAse Diagrams) method was adopted to perform the nonequilibrium simulation using the Thermo-Calc Software Version 2017a. On the basis of the Thermo-Calc HEA thermodynamic database (TCHEA2), the Scheil-Gulliver model (*38*, *39*) was applied to simulate the nonequilibrium-localized fcc phase region, where γ′ and γ* nanoprecipitates could coexist as a hierarchical structure in the fcc γ matrix.

## Supplementary Material

http://advances.sciencemag.org/cgi/content/full/4/10/eaat8712/DC1

## SUPPLEMENTARY MATERIALS

Supplementary material for this article is available at http://advances.sciencemag.org/cgi/content/full/4/10/eaat8712/DC1 Alloy design strategy Fig. S1. TEM of the bulk Fe_25_Co_25_Ni_25_Al_10_Ti_15_ HEA. Fig. S2. Twins in the primary fcc phase. Fig. S3. HRTEM images of hierarchical precipitates. Fig. S4. Sequential snapshots from a video recorded during the in situ TEM compression test. Fig. S5. Fracture morphology after in situ SEM microtensile testing. Fig. S6. X-ray scattering and microstructure of the Fe_25_Co_25_Ni_25_Al_10_Ti_15_ HEA powders. Fig. S7. The Scheil-Gulliver simulation of the nonequilibrium fcc phase region. Fig. S8. SEM micrograph of the microforce sensor’s flat probe tip with custom-milled tensile grip geometry. Table S1. EDS/TEM and EDS/STEM results of the phases in the bulk Fe_25_Co_25_Ni_25_Al_10_Ti_15_. Table S2. Processing routes and microstructures of selected HEAs. Movie S1. During the initial deformation, dislocations were first generated in the γ matrix, and as the displacement increased, dislocations sheared the hierarchical γ′ and γ* precipitates.
