# Supplementary material for: A high-entropy alloy with hierarchical nanoprecipitates and ultrahigh strength
Source: Sci Adv. 2018 Oct 12;4(10):eaat8712. doi: 10.1126/sciadv.aat8712 (PMC6184785; doi:10.1126/sciadv.aat8712)
Supplement: http://advances.sciencemag.org/cgi/content/full/4/10/eaat8712/DC1 [file supp_4_10_eaat8712__index.html]

Science Advances | Science Advances

## Supplementary Materials

**The PDF file includes:**

- Alloy design strategy
- Fig. S1. TEM of the bulk Fe25Co25Ni25Al10Ti15 HEA.
- Fig. S2. Twins in the primary fcc phase.
- Fig. S3. HRTEM images of hierarchical precipitates.
- Fig. S4. Sequential snapshots from a video recorded during the in situ TEM compression test.
- Fig. S5. Fracture morphology after in situ SEM microtensile testing.
- Fig. S6. X-ray scattering and microstructure of the Fe25Co25Ni25Al10Ti15 HEA powders.
- Fig. S7. The Scheil-Gulliver simulation of the nonequilibrium fcc phase region.
- Fig. S8. SEM micrograph of the microforce sensor’s flat probe tip with custom-milled tensile grip geometry.
- Table S1. EDS/TEM and EDS/STEM results of the phases in the bulk Fe25Co25Ni25Al10Ti15.
- Table S2. Processing routes and microstructures of selected HEAs.

Download PDF

**Other Supplementary Material for this manuscript includes the following:**

- Movie S1 (.mp4 format). During the initial deformation, dislocations were first generated in the γ matrix, and as the displacement increased, dislocations sheared the hierarchical γ′ and γ\* precipitates.

**Files in this Data Supplement:**

- Adobe PDF - aat8712\_SM.pdf
