# Supplementary material for: A high-entropy alloy with hierarchical nanoprecipitates and ultrahigh strength
Source: Sci Adv. 2018 Oct 12;4(10):eaat8712. doi: 10.1126/sciadv.aat8712 (PMC6184785; doi:10.1126/sciadv.aat8712)
Supplement: http://advances.sciencemag.org/cgi/content/full/4/10/eaat8712/DC1 [file aat8712_SM.pdf]

Science Advances
AAAS

advances.sciencemag.org/cgi/content/full/4/10/eaat8712/DC1

Supplementary Materials for

A high-entropy alloy with hierarchical nanoprecipitates and ultrahigh strength

Zhiqiang Fu, Lin Jiang, Jenna L. Wardini, Benjamin E. MacDonald, Haiming Wen, Wei Xiong, Dalong Zhang, Yizhang Zhou, Timothy J. Rupert, Weiping Chen*, Enrique J. Lavernia*

*Corresponding author. Email: mewpchen@scut.edu.cn (W.C.); lavernia@uci.edu (E.J.L.)

Published 12 October 2018, *Sci. Adv.* **4**, eaat8712 (2018)
DOI: 10.1126/sciadv.aat8712

The PDF file includes:

Alloy design strategy
Fig. S1. TEM of the bulk $Fe_{25}Co_{25}Ni_{25}Al_{10}Ti_{15}$ HEA.
Fig. S2. Twins in the primary fcc phase.
Fig. S3. HRTEM images of hierarchical precipitates.
Fig. S4. Sequential snapshots from a video recorded during the in situ TEM compression test.
Fig. S5. Fracture morphology after in situ SEM microtensile testing.
Fig. S6. X-ray scattering and microstructure of the $Fe_{25}Co_{25}Ni_{25}Al_{10}Ti_{15}$ HEA powders.
Fig. S7. The Scheil-Gulliver simulation of the nonequilibrium fcc phase region.
Fig. S8. SEM micrograph of the microforce sensor's flat probe tip with custom-milled tensile grip geometry.
Table S1. EDS/TEM and EDS/STEM results of the phases in the bulk $Fe_{25}Co_{25}Ni_{25}Al_{10}Ti_{15}$.
Table S2. Processing routes and microstructures of selected HEAs.

Other Supplementary Material for this manuscript includes the following:

(available at advances.sciencemag.org/cgi/content/full/4/10/eaat8712/DC1)

Movie S1 (.mp4 format). During the initial deformation, dislocations were first generated in the γ matrix, and as the displacement increased, dislocations sheared the hierarchical γ′ and γ* precipitates.

Alloy design strategy

In the present work, we designed the $Fe_{25}Co_{25}Ni_{25}Al_{10}Ti_{15}$ HEA based on the fcc single-phase equiatomic FeCoNi medium-entropy alloy (MEA) in which two elements Al and Ti were added. The reactive Al and Ti have good affinity with each other. Furthermore, the enthalpies of mixing for different atom-pairs among Al, Fe, Co, Ni and Ti, Fe, Co, Ni are similarly negative (*33*). It appears that Al and Ti have similar ability to form intermetallics (precipitates) with Fe, Co and Ni, thereby leading to a trend of forming precipitates with multi-principal elements. The empirical criteria, $\delta \leq 6.6\%$ and $\Omega = \frac{T_m \Delta S_{mix}}{|\Delta H_{mix}|} \geq 1.1$, have been widely used to predict stable solid solutions would form in HEAs (*32*). Hence, they are utilized to optimize the contents of Al and Ti which are to be added into the fcc single-phase equiatomic FeCoNi MEA. Herein the parameters ΔH_{mix} (enthalpy of mixing), ΔS_{mix} (entropy of mixing), δ (atomic difference) and T_m are defined as follows (*32*)

$$\Delta H_{mix} = \sum_{i=1, i\neq j}^{n} \Omega_{ij} c_i c_j = \sum_{i=1, i\neq j}^{n} 4H_{ij}^{mix} c_i c_j \tag{1}$$

$$\Delta S_{mix} = -R\sum_{i=1}^{n} (c_i \ln c_i),\ \sum\nolimits_{i=1}^{n} c_i = 1 \tag{2}$$

$$\delta = \sqrt{\sum_{i=1}^{n} c_i (1 - r_i / \bar{r})^2}\ ,\ \bar{r} = \sum\nolimits_{i=1}^{n} c_i r_i \tag{3}$$

$$T_{\rm m} = \sum_{i=1}^{n} c_{\rm i} (T_m)_i \tag{4}$$

Where $(T_m)_i$ is the melting point of the ith component and R represents the gas constant (8.314 $JK^{-1}mol^{-1}$); H_{ij}^{mix} is the enthalpy of mixing of the ith and j^{th} components based on the Miedema macroscopic model (*33*). In addition, c_i and c_j are the atomic percentages of the ith and j^{th} constituent elements; and r_i indicates the atomic radius of the ith component (*32*).

To ensure the formation of stabilized solid-solution phases without additional complex phases, the empirical conditions of $\Omega \geq 1.1$ and $\delta \leq 6.6\%$ should be satisfied at minimum. While values of Ω above 1.1 have proven to yield complex multi-phase alloys, we also hypothesized that when Ω infinitely approaches the threshold value of 1.1, the nature of secondary phases that form may be beneficial to the structural performance of the alloy (i.e. coherent precipitates). Accordingly, 10 at.% Al and 15 at.% Ti have been added to the fcc single-phase FeCoNi prototype alloy, leading to $\Omega = 1.119$ and $\delta = 6.277$. Therefore, the alloy with the chemical composition of $Fe_{25}Co_{25}Ni_{25}Al_{10}Ti_{15}$ (at.%) has been successfully designed.

In summary, the strategy can be summarized as follows: (1) Initially, we selected a single-phase fcc MEA as the prototype alloy, and the Al and Ti elements are both introduced into the prototype alloy; (2) The empirical conditions of $\Omega \geq 1.1$ and $\delta \leq 6.6\%$ should be satisfied at a minimum with the aim to control the addition concentrations of Al and Ti; (3) Meanwhile, Ω should approach the threshold value of 1.1 in an attempt to develop an alloy with beneficial secondary phases.

Supplementary Figure 1

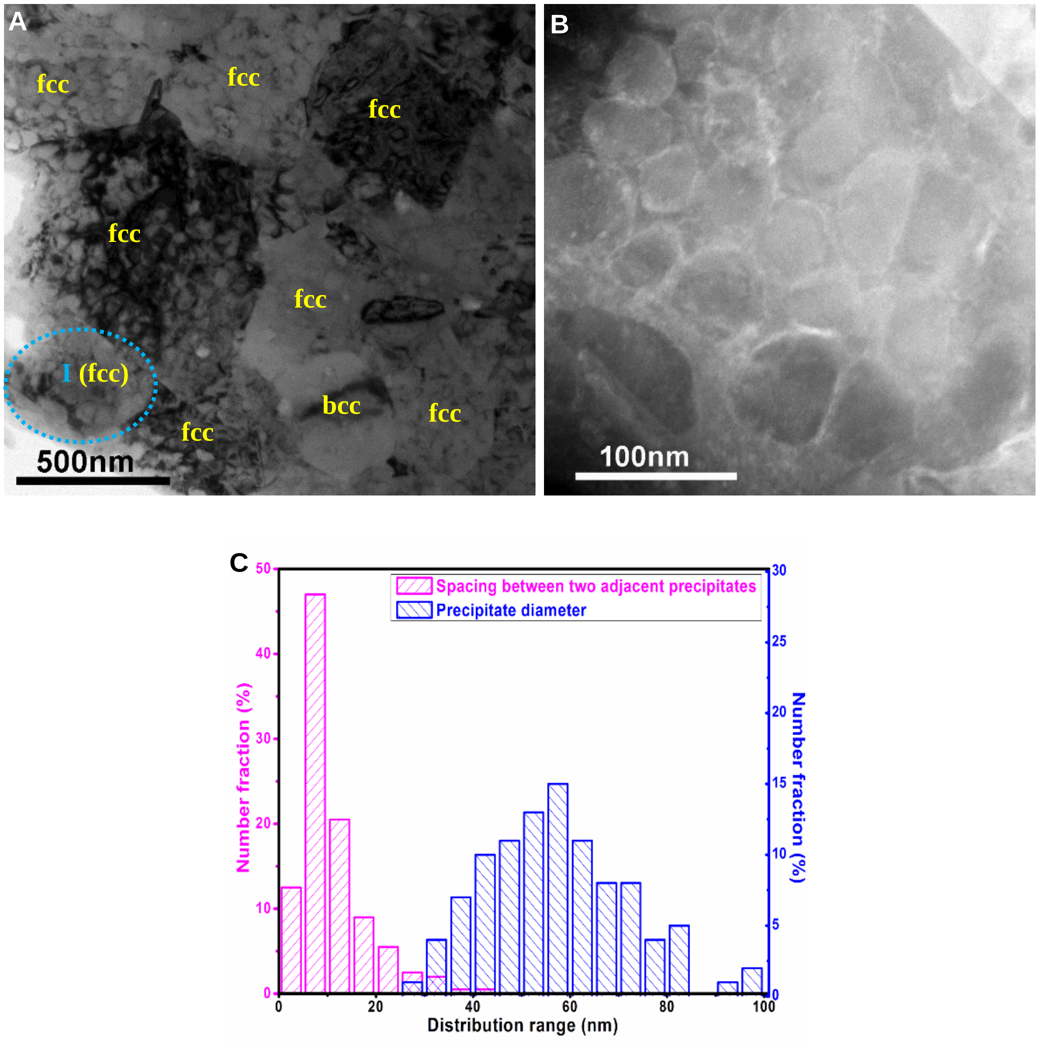

Fig. S1. TEM of the bulk $Fe_{25}Co_{25}Ni_{25}Al_{10}Ti_{15}$ HEA. (**A**) Bright-field TEM image. (**B**) STEM image of grain I in (A) confirming the presence of precipitates inside the fcc grains. (**C**) Statistical distributions of the spacing between two adjacent γ' precipitates as well as their diameters, showing that the average spacing between adjacent γ' precipitates is 11 nm and their average diameter is 57 nm.

Supplementary Figure 2

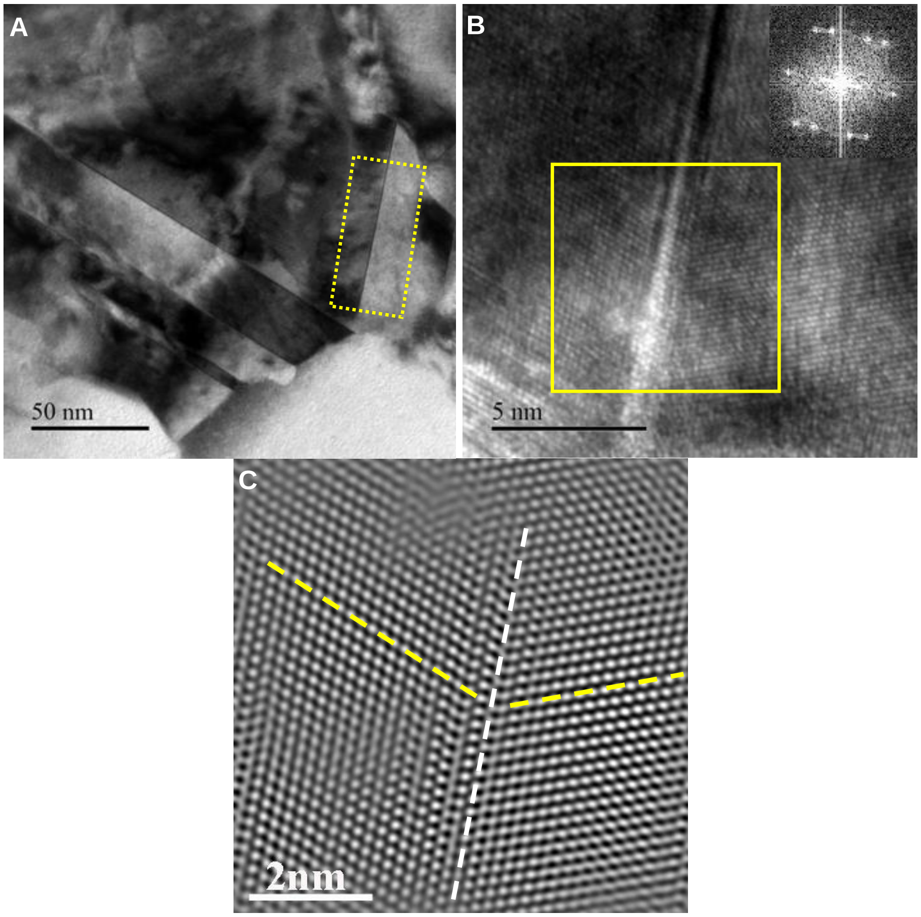

Fig. S2. Twins in the primary fcc phase. (**A**) Bright-field TEM image of an fcc phase. (**B**) HRTEM image of the rectangular region in (A) where the inset FFT corresponds to the boxed region in (B). (**C**) IFFT of the inset FFT in (B), showing the presence of twins.

Supplementary Figure 3

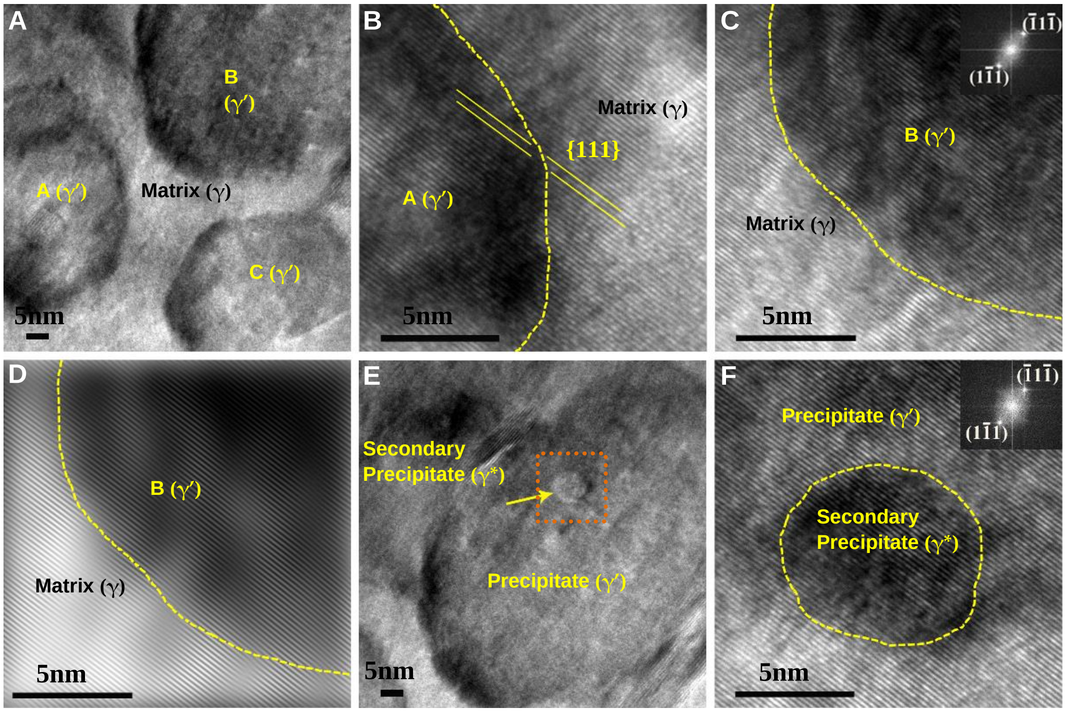

Fig. S3. HRTEM images of hierarchical precipitates. (**A**) HRTEM image of three γ' precipitates and the γ matrix. (**B**) HRTEM image of the γ matrix and γ' precipitate A in (A). (**C**) HRTEM image of the γ matrix and γ' precipitate B in (A) with corresponding FFT presented in the inset. (**D**) IFFT of (C). (**E**) Bright-field TEM image of a γ' precipitate containing some γ* secondary precipitates. (**F**) HRTEM image of the square area in (E) with corresponding FFT presented in the inset.

Supplementary Figure 4

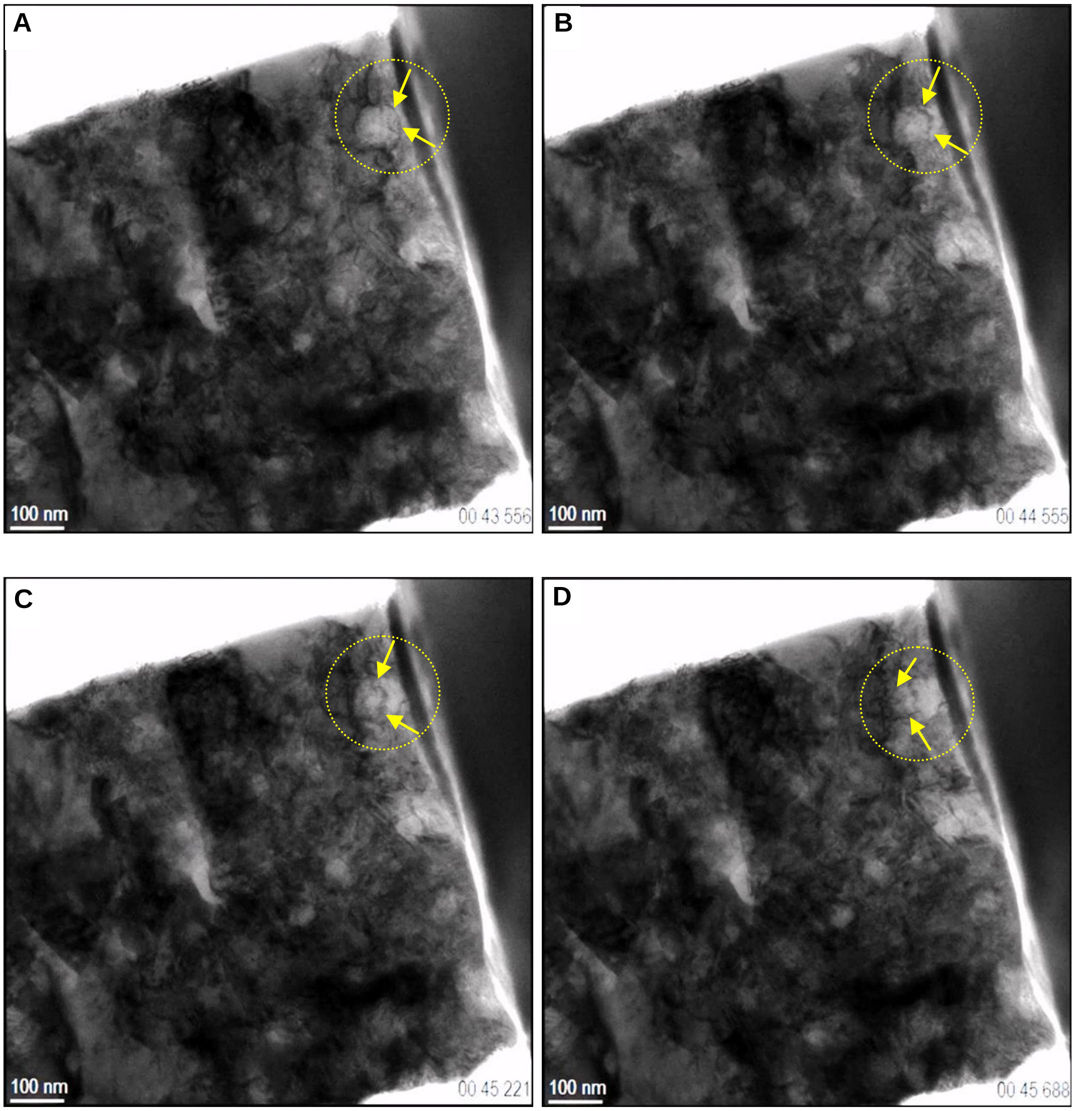

Fig. S4. Sequential snapshots from a video recorded during the in situ TEM compression test. The arrows follow dislocations as they pass through a γ’ nano-precipitate within the γ matrix, implying the nano-precipitate is being sheared.

Supplementary Figure 5

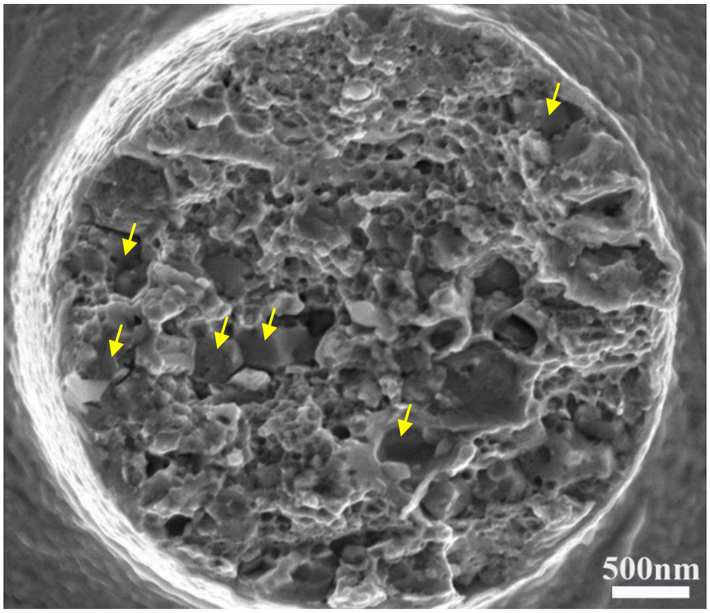

Fig. S5. Fracture morphology after in situ SEM microtensile testing. Faceted regions indicated by arrows corresponding to brittle failure, and dimpled regions corresponding to ductile failure.

Supplementary Figure 6

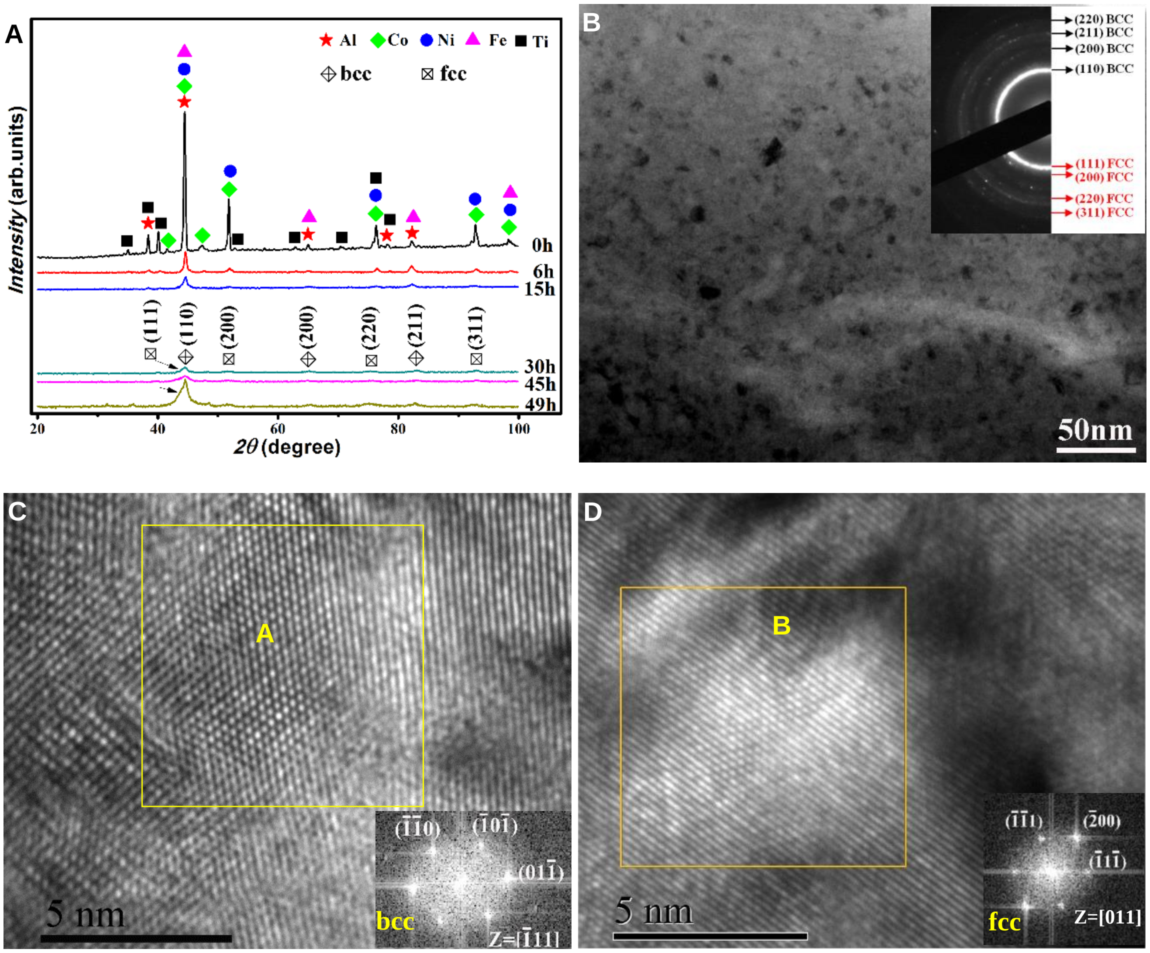

Fig. S6. X-ray scattering and microstructure of the $Fe_{25}Co_{25}Ni_{25}Al_{10}Ti_{15}$ HEA powders. (**A**) XRD patterns of the $Fe_{25}Co_{25}Ni_{25}Al_{10}Ti_{15}$ HEA powders with different milling times. (**B**) Bright-field TEM image of the 49 h milled powders with corresponding SAED pattern presented in the inset. (**C**) HRTEM image of the bcc phase with SAED pattern corresponding to region A presented in the inset. (**D**) HRTEM image of the fcc phase with SAED pattern corresponding to region B presented in the inset, indicating a disordered fcc structure.

Supplementary Figure 7

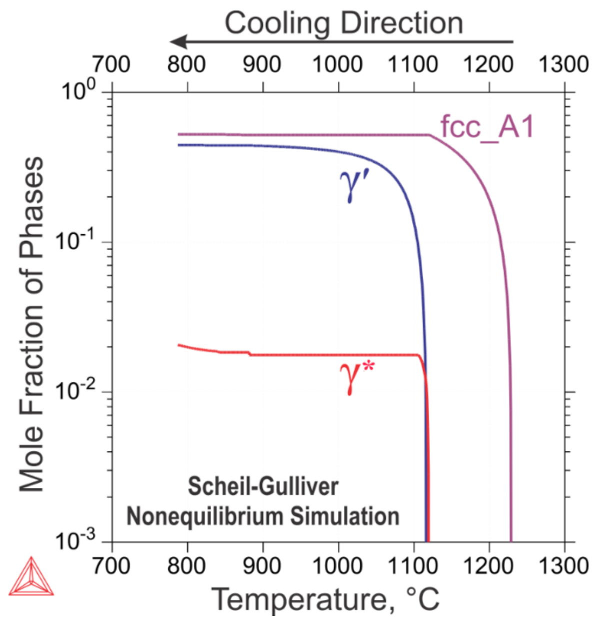

Fig. S7. The Scheil-Gulliver simulation of the nonequilibrium fcc phase region. The composition input of the simulation is $Co_{27.0}Ni_{27.4}Fe_{29.8}Al_{3.5}Ti_{12.3}$ determined by EDS/TEM (see Table S1). This explains the observation of the (γ+γ'+γ*) three phase region in the as-fabricated microstructure. The composition of the γ* phase is rich in Fe with a composition of $Fe_{47.22}Ti_{26.23}Co_{22.32}Ni_{4.22}Al_{0.01}$ at 787 °C as the lowest formation temperature in the cooling process.

Supplementary Figure 8

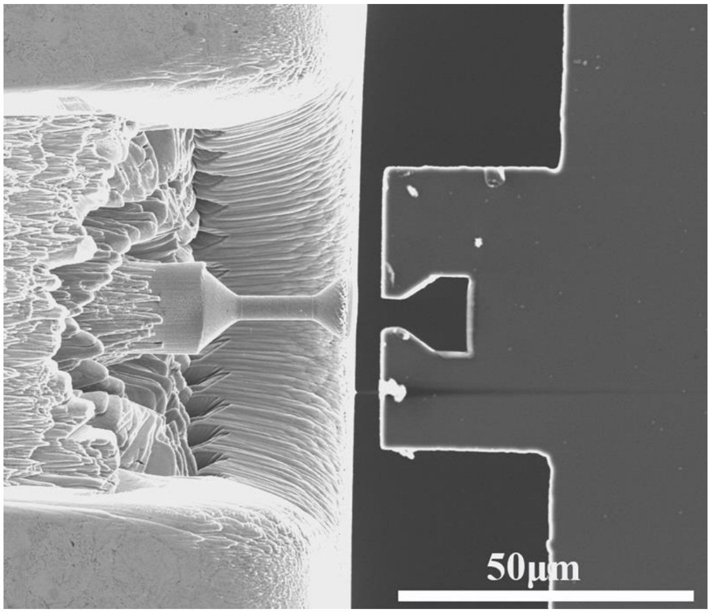

Fig. S8. SEM micrograph of the microforce sensor's flat probe tip with custom-milled tensile grip geometry.

Supplementary Tables

Table S1. EDS/TEM and EDS/STEM results of the phases in the bulk $Fe_{25}Co_{25}Ni_{25}Al_{10}Ti_{15}$.

Phase (Structure)	Co	Ni	Fe	Al	Ti
Nominal composition	25	25	25	10	15
bcc phase (B2)	27.18±0.48	25.48±0.33	13.4±0.29	13.04±0.81	20.90±0.14
fcc phase (γ+γ'+γ* overall, $L1_2$)	27.02±0.55	27.38±1.43	29.78±2.30	3.50±0.59	12.32±1.72
fcc matrix, γ (A1)	28.27±1.27	23.27±4.68	44.43±3.62	1.73±2.19	2.3±2.76
Precipitate, γ' ($L1_2$)	25.68±1.71	39.04±3.27	15.17±1.46	2.34±2.21	17.77±2.00

Table S2. Processing routes and microstructures of selected HEAs.

Alloys	Processing route	Microstructure	Refs.
HfNbTaTiZr	CR*	bcc	(*19*)
$AlCoCrFeNi_{2.1}$	CR	nanostructured $L1_2$+B2	(*20*,*21*)
$AlCoCrFeNi_{2.1}$	cryo-rolling + annealing	nanostructured $L1_2$+B2	(*22*)
CoCrFeNiMn	HPT*	nanostructured fcc	(*23*)
CoCrFeNiMn	HPT + annealing	fcc+bcc+σ	(*23*)
FeNiCoCu	HPT	nanostructured fcc+bcc	(*24*)
$(FeNiCoCu)_{86}$-Ti_7Al_7	HPT	nanostructured fcc	(*24*)
TiZrNbHfTa	HPT	nanostructured bcc	(*25*)
TiZrNbHfTa	HPT + annealing	nanostructured bcc+hcp	(*25*)
$Cr_{15}Fe_{20}Co_{35}Ni_{20}Mo_{10}$	CR + annealing	fcc matrix + nanoprecipitates	(*26*)
$(FeCoNiCr)_{94}Ti_2Al_4$	CR + annealing	fcc matrix + nanoprecipitates	(*28*)
TaHfZrTi	casting	bcc	(*29*)
$Ni_{1.5}Co_{1.5}CrFeTi_{0.5}$	MA+SPS*	fcc+nanosized oxides	(*30*)
$Fe_{25}Co_{25}Ni_{25}Al_{10}Ti_{15}$	MA+SPS	fcc+ hierarchical nanoprecipitates+B2	This work

* CR: cold rolling; HPT: high pressure torsion; MA+SPS: mechanical alloying and spark plasma sintering.
